# Clinical Efficacy and Safety of Qishen Yiqi Dropping Pill Combined with Conventional Western Medicine in the Treatment of Chronic Heart Failure: A Systematic Review and Meta-Analysis

**DOI:** 10.1155/2021/6612653

**Published:** 2021-02-02

**Authors:** Lisheng Chen, Ruilin Wang, Honghong Liu, Shizhang Wei, Manyi Jing, Min Wang, Yanling Zhao

**Affiliations:** ^1^Department of Pharmacy, Fifth Medical Center, General Hospital of Chinese PLA, Beijing, China; ^2^Department of Pharmacy, Hebei North University, Zhangjiakou, China; ^3^Department of Traditional Chinese Medicine, Fifth Medical Center, General Hospital of Chinese PLA, Beijing, China; ^4^Integrative Medical Center, Fifth Medical Center, General Hospital of Chinese PLA, Beijing, China

## Abstract

**Objective:**

The systematic review was designed to evaluate the safety and efficacy of Qishen Yiqi dropping pill combined with conventional Western medicine in the treatment of chronic heart failure (CHF).

**Methods:**

Relevant randomized controlled trials (RCTs) investigating the clinical efficacy of Qishen Yiqi dropping pill combined with conventional Western medicine in treating CHF were widely searched in electronic databases, including PubMed, Cochrane Library, EMBASE, CBM, CNKI, Read-show database, VIP database, and WanFang up to December 26, 2020. The methodological quality of each trial was assessed according to the Cochrane Reviewers' Handbook 5.0. Meta-analysis was performed by using Review Manager 5.3.

**Results:**

Twenty-one RCTs (*N* = 2162) that met the criteria were included in the review for the assessment of methodological quality. Meta-analysis showed that compared with the conventional Western medicine (control group), Qishen Yiqi dropping pill combined with conventional Western medicine (experience group) significantly improved clinical efficiency, left ventricular end-diastolic diameter (LVEDD), left ventricular end-systolic diameter (LVESD), left ventricular ejection fraction (LVEF), brain natriuretic peptide level (BNP), 6 min-walk distance (6-MWD), and adverse reactions.

**Conclusion:**

Qishen Yiqi dropping pill combined with conventional Western medicine are better than conventional Western medicine alone to improve the indicators of patients with CHF, which provides a certain basis for the treatment of CHF.

## 1. Introduction

Heart failure (HF) is a complex set of clinical syndromes, and the main clinical manifestations of which are ventricular filling and ejection function impairment caused by various cardiac structural or functional diseases, insufficient blood perfusion in organs and tissues, and insufficient cardiac output to meet the needs of body tissue metabolism [1]. HF is a serious or terminal stage of various heart diseases [2]. The clinical therapeutic effect of HF is limited, and the 5-year fatality rate is high. Due to the increasing incidence of chronic heart failure (CHF) year by year, the main causes of cardiovascular diseases such as coronary heart disease and hypertension have become a major public health problem in the world. Because the poor prognosis and high mortality have caused serious damage to the health of the people and increased the economic burden on patients and society [3]. The ultimate goal of treatment for chronic heart failure is to extend patient survival, reduce patient pain, improve quality of life, minimize hospitalization and mortality, and prevent complications. Therefore, the treatment of chronic heart failure should adopt the corresponding comprehensive measures. With the continuous development of modern medicine, all kinds of treatment methods are changing with each passing day, and many technologies are not mature. In addition, the treatment effect is limited, and the treatment cost is high; traditional Chinese medicine treatment is still the mainstream [4].

CHF belongs to the category of “chest paralysis, palpitation, edema, panting syndrome.” The pathogenesis of CHF is the deficiency of the essence and the heart and kidney (Yang) and the stagnation of blood stasis [5]. Qi deficiency and blood stasis are the basic pathogenesis of heart failure, which runs through the whole process of the disease. It has been demonstrated that pathogenic factors are qi deficiency, yin deficiency, yang deficiency, blood stasis, phlegm, and so on, and the most common symptoms are fatigue, edema, palpitations, and gasping [6]. The therapeutic principles of chronic heart failure in the field of traditional Chinese medicine are to benefit the heart Qi, warm the heart Yang, and invigorate the heart blood [7]. Although Western medicine is effective, it still cannot solve the accompanying symptoms of heart failure such as asthenia and abdominal distention, and long-term use of Western medicine will have toxic side effects. Traditional Chinese medicine treatment can effectively improve the main symptoms and concomitant symptoms of patients. In recent years, domestic scholars of traditional Chinese medicine and integrated traditional Chinese and Western medicine have carried out a lot of research work on the treatment plan of traditional Chinese medicine, clinical efficacy evaluation, and the safety of combined use of Chinese and Western medicine for CHF.

**Figure 1:**
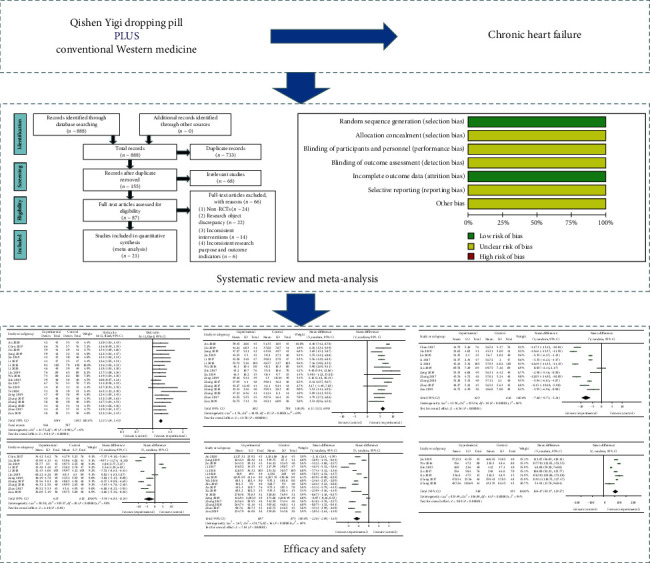
Research strategy of the current study.

Traditional Chinese medicine (TCM), with its unique curative effect in heart failure treatment, is gaining increasing attention as the discovery of novel antiheart failure drugs has become the pursuit of pharmaceutical. Qishen Yiqi dropping pill is one of the representative traditional Chinese medicine preparations, which is composed of *Astragalus*, *Salvia miltiorrhiza*, *Panax notoginseng,* and deodorized oil. Modern pharmacological research shows that it has the effects of delaying ventricular reconstruction, controlling ventricular rate [8, 9], antiplatelet aggregation, promoting angiogenesis, and has a good therapeutic effect on the myocardial ischemia-reperfusion injury and inflammation. Qishen Yiqi dropping pill is widely used in the treatment of chronic heart failure and coronary heart disease, but there is no systematic evaluation report on the outcome of it in the treatment of CHF. Therefore, in order to promote the rational use of Qishen Yiqi dropping pill in clinical practice, this study adopts the method of randomized controlled tests to systematically evaluate the safety and effectiveness of Qishen Yiqi dropping pill combined with conventional Western medicine in the treatment of CHF (Figure 1).

## 2. Materials and Methods

### 2.1. Ethics Approval and Consent to Participate

Due to this study does not involve animal and patient experiments, the ethics approval and consent to participate are not applicable.

### 2.2. Inclusion Criteria and Exclusion Criteria

#### 2.2.1. Study Type

The randomized controlled trials (RCTs) of Qishen Yiqi dropping pills in the treatment of chronic heart failure at home and abroad had similar research methods and complete general data, which were statistically based on unified indicators.

#### 2.2.2. Study Object

The diagnostic criteria of chronic heart failure referred to the guideline for diagnosis and treatment of chronic heart failure [10], and the indicators of patients were comparable.

#### 2.2.3. Intervention Measures

The control groups were treated with conventional Western medicine (according to the guidelines of chronic heart failure [11], including diuretics, *β*-blockers, nitrates, digitalis, aldosterone receptor antagonists, ACEI, or ARB), and the group of experimental groups was treated with Qishen Yiqi dropping pill on the basis of conventional Western medicine.

#### 2.2.4. Exclusion Criteria

1. Descriptive study only and no clinical control trials
2. The control group received other treatments besides the routine basic treatment
3. The intervention measures in the Qishen Yiqi dropping pill group were not only Qishen Yiqi dropping pills, but also other treatment methods not used in the control group
4. Repeated reports or studies with inaccurate or incomplete literature data
5. Individual cases or empirical reports, animal experiments, and reviews
6. Cannot reflect the research on the clinical effect of Qishen Yiqi dropping pill
7. Outcome indicators are inconsistent

#### 2.2.5. Outcome Indicators

In this systematic review and meta-analysis, the outcome indicators were clinical efficacy and safety of Qishen Yiqi dropping pill combined with conventional Western medicine, which were clinically relevant when evaluating the pharmacology of Qishen Yiqi dropping pill in relation to the probable mechanisms. According to the guiding principles for clinical research of new drugs of traditional Chinese medicine [12] and New York Heart Association (NYHA) classification to formulate efficacy evaluation criteria: ① clinical efficacy is defined on 3 levels: markedly effective rate: patients achieve complete remission or cardiac function improves above level II; effective rate: patients achieve partial remission or cardiac function improves to level I. Signs and symptoms are relieved to a certain degree; ineffective rate: patient with cardiac insufficiency improves to level I, or signs and symptoms are not significantly improved. ② LVESD, ③ LVEDD, ④ LVEF, ⑤ BNP, ⑥ NT-proBNP, ⑦ 6-MWD, ⑧ other indicators, and ⑨ adverse reactions.

### 2.3. Search Strategy

A comprehensive systematic search concerning the clinical efficacy and safety of Qishen Yiqi dropping pill combined with conventional Western medicine in treating CHF was performed to identify the published RCTs from inception to December 26, 2020. The databases included PubMed, Cochrane Library, EMBASE, CBM, CNKI, Read-show database, VIP database, and WanFang. The following search terms were used: “Qishenyiqi dropping pill” [Mesh terms] OR “Qishenyiqi” [Mesh terms] AND “heart failure” [Mesh terms] OR “chronic heart failure” [Mesh terms]. The involved studies were downloaded for further evaluation. All unclear questions were addressed by contacting the study authors by e-mail.

### 2.4. Article Selection and Data Extraction

Relevant studies were detected in light of the search terms. We followed the methods of Wang et al. 2017 [13]. According to the exclusion and inclusion criteria, two researchers read the title and abstract of studies independently and then excluded the studies that obviously do not meet the inclusion criteria. Carefully read the full text of the studies that may meet the inclusion criteria to determine whether it meets the inclusion criteria and then cross-check. The contents extracted included the first author of the study, the year of the paper, the intervention method, the sample size of the experimental group and the control group, the course of treatment, the dose, the clinical efficacy, the improvement indexes of cardiac function, and the adverse reactions. When problems or opinions are not unified, they shall be solved through discussion or consultation with a third party.

### 2.5. Quality Evaluation

The methodological quality assessment was carried out using the Cochrane Handbook for Systematic Reviews of Interventions [14]. Seven domains including random sequence generation (selection bias), allocation concealment (selection bias), blinding of participants and personnel (performance bias), blinding of outcome assessment (detection bias), incomplete outcome data (attrition bias), selective reporting (reporting bias), and other bias were used for the methodological quality of each included trials. For all the relevant outcomes in the relevant domains, the quality of each item was classified using a nominal scale: low risk of bias, high risk of bias, or unclear risk of bias.

### 2.6. Statistical Analysis

The statistical analysis was performed by Review Manager 5.3 software (the Cochrane Collaboration, Copenhagen, and the Nordic Cochrane Centre). For measurement data, dichotomous variables were presented as risk ratio (RR), while continuous outcomes were presented as the mean difference (MD) or standard mean difference (SMD) with 95% confidence intervals (CIs). The *I*-square (*I*^2^) statistic was used to assess heterogeneity. If *p* > 0.1, *I*^2^ < 50%, indicating small heterogeneity; the fixed effect model was used for meta-analysis. If *I*^2^ > 50%, *p* < 0.1, it indicated that there was a high degree of heterogeneity among the study results; then, the random effect model was applied. The source of heterogeneity was analyzed by subgroup analysis. Sensitivity analysis was used for the stability of the analysis results. Whether bias occurs or not was indicated by the funnel plot.

## 3. Results

### 3.1. Identification of Included Studies

**Figure 2:**
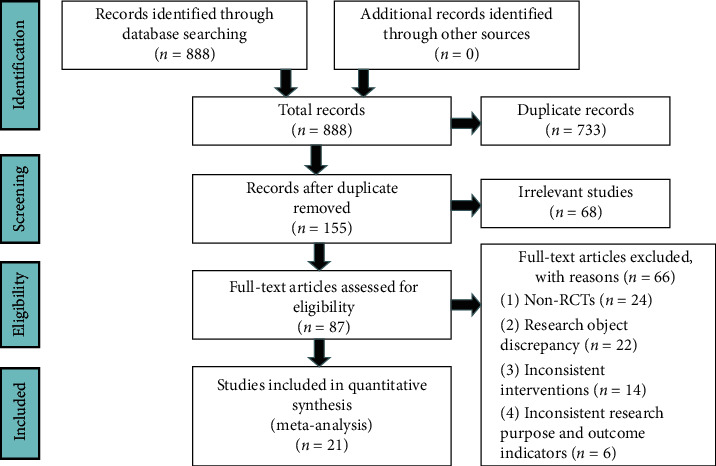
Flow diagram of study selection.

A total of 888 relevant articles were found by computer preliminary inspection, and 155 articles were eventually selected for further screening after duplicate checking. The rest of the articles were carefully screened by reference to the exclusion and inclusion criteria, including 24 animal experiments, 17 reviews, and 27 systematic evaluations and meta-analyses, and 87 full articles were used for further assessment. Among the objects, non-RCTs (*n* = 24), research object discrepancy (*n* = 22), inconsistent interventions (*n* = 14), and inconsistent research purposes and outcome indicators (*n* = 6) were excluded. Finally, 21 studies [15–35] with 2162 patients with CHF who met the criteria were included in the meta-analysis. The flow diagram of the study screening is shown in Figure 2. The characteristics of the included studies are shown in Table 1.

### 3.2. Quality Evaluation of Included Studies

**Figure 3:**
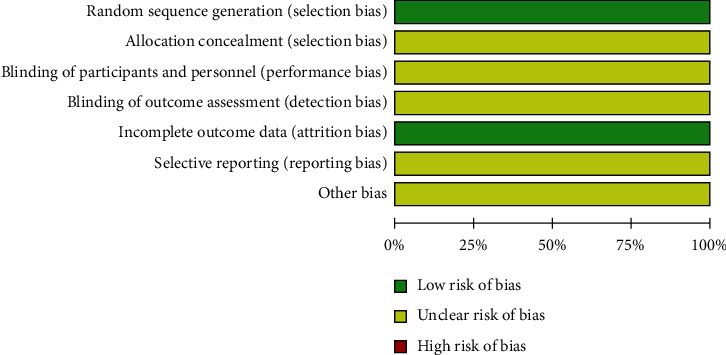
Risk of bias in these included trails.

The methodological quality for each included study was evaluated according to the Cochrane risk of bias estimation. In terms of random sequence generation, all the included trials were RCTs, and they were designated as low risk. On the aspects of allocation concealment, blinding of participants and personnel, and blinding of outcome assessment, all the trails were not mentioned clearly. In terms of incomplete outcome data, 21 studies [15–35] were not reported on selective reporting. None of the studies reported other biases. The quality evaluation of the included studies is shown in Figure 3.

### 3.3. Clinical Efficacy

**Figure 4:**
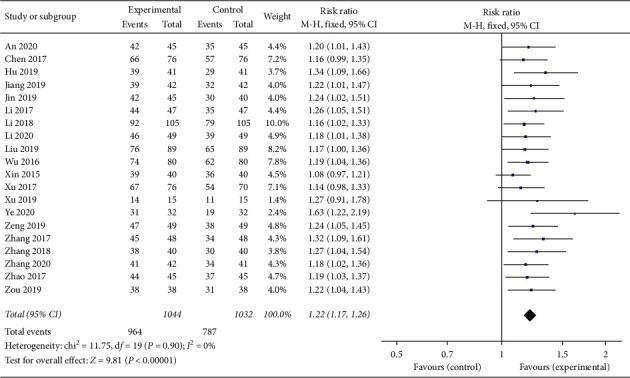
Forest plot of clinical efficacy rate comparing the experimental group and control group.

Twenty trials [15–27, 29–35] (*N* = 2076) provided data comparing the clinical efficacy between the experimental groups with 1044 patients and control groups with 1032 patients. The test of heterogeneity results showed that there was homogeneity among the studies (*p*=0.90, *I*^2^=0%), so the fixed effect model was used for analysis. Meta-analysis results showed that the experimental groups were associated with a relatively greater improvement in the total efficacy rate in the treatment of CHF (RR = 1.21, 95% CI (1.17, 1.26), *p* < 0.00001) (Figure 4).

### 3.4. LVEF Improvement

**Figure 5:**
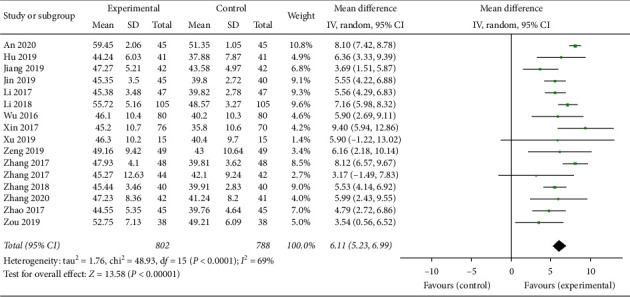
Forest plot of LVEF comparing the experimental group and control group.

In this study, a total of 16 trials [16–20, 22, 24–31, 33, 35] with 1590 patients investigated measurements of LVEF between the experimental and control groups. There were 802 patients in experimental groups and 788 patients in control groups. A random effect model was used to pool this meta-analysis (*p* < 0.0001, *I*^2^ = 69%). As shown in Figure 5, the result showed the increase in LVEF was significantly better in the experimental groups than in the control groups (MD = 6.11, 95% CI (5.23, 6.99), *p* < 0.00001).

### 3.5. The Decrease of LVEDD

**Figure 6:**
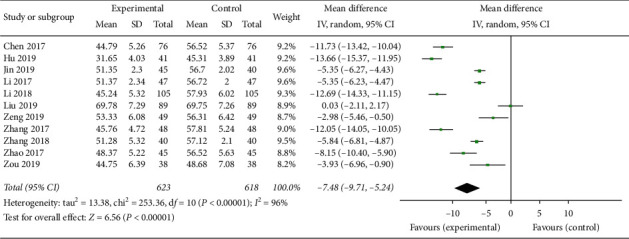
Forest plot of LVEDD comparing the experimental group and control group.

In the study, a total of 11 articles [15, 16, 18–21, 26, 28–31] with 1241 patients including 623 patients in experimental groups and 618 patients in control groups assessed the index of LVEDD. A random effect model was performed for analysis (*p* < 0.00001, *I*^2^ = 96%). The results showed that the experimental groups were superior to the control groups in reducing the LVEDD (MD = −7.48, 95% CI (−9.71, −5.24), *p* < 0.00001) (Figure 6).

### 3.6. The Decrease of LVESD

**Figure 7:**
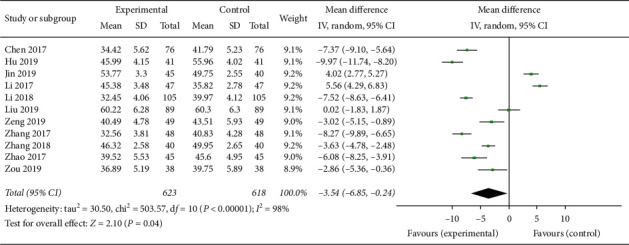
Forest plot of LVESD comparing the experimental group and control group.

A total of 11 articles [15, 16, 18–21, 26, 28–31] with 1241 patients, 623 patients in experimental groups and 618 patients in control groups, assessed the index of LVEDD between the experimental and control groups. There was heterogeneity between groups (*p* < 0.00001, *I*^2^ = 98%), so the random effect model was used for analysis. As shown in Figure 7, the pooled analysis showed that the decrease of experimental groups on LVESD of patients with chronic heart failure was more than that of the control groups (MD = −3.54, 95% CI (−6.85, −0.24), *p* < 0.05).

### 3.7. BNP Reduction

**Figure 8:**
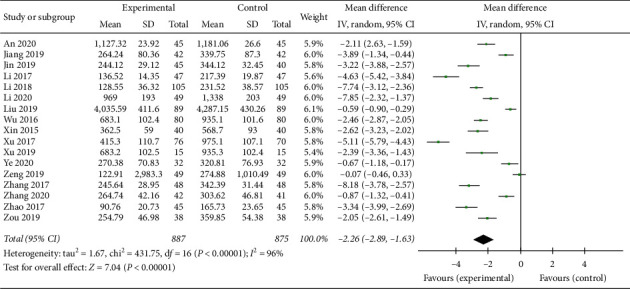
Forest plot of BNP comparing the experimental group and control group.

Seventeen studies [17–26, 29–35] with 1762 subjects, 887 cases in experimental groups and 875 cases in control groups, reported the measurements of BNP. There was heterogeneity of the index, and the random effect model was performed for analysis (*p* < 0.00001, *I*^2^ = 96%). The results showed that experimental groups significantly decreased BNP (SMD = −2.26, 95% CI (−2.89, −1.63), *p* < 0.00001) compared with control groups (Figure 8).

### 3.8. The Improvement of 6-MWD

**Figure 9:**
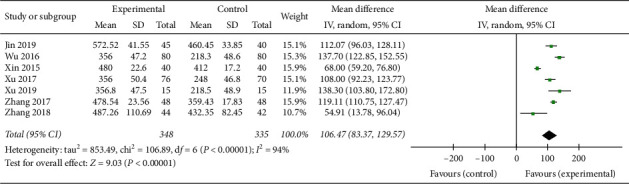
Forest plot of 6-MWD comparing the experimental group and control group.

In this systematic review, a total of 7 studies [18, 22–25, 28, 29] with 683 patients including 348 patients of experimental groups and 335 patients of control groups evaluated the level of 6-MWD. A random effect model was conducted for analysis according to the heterogeneity test among each trial (*p* < 0.00001, *I*^2^ = 94%). The results suggested that the experimental groups improved 6-MWD compared with the control groups (MD = 106.47, 95% CI (83.37, 129.57), *p* < 0.00001), and the exercise endurance of CHF patients was significantly increased (Figure 9).

### 3.9. Adverse Reactions

A total of 15 studies [15, 16, 18, 19, 21–24, 26, 27, 29–32, 35] reported adverse reactions as outcome indicators, among which 8 studies [15, 16, 18, 19, 23, 27, 29, 33] mentioned no serious adverse reactions and the remaining 7 studies [21, 22, 24, 26, 30, 31, 35] reported in detail adverse reactions during the treatment, as shown in Table 2. The main adverse reactions included hypotension [21, 26, 31, 35], dry cough [22, 24, 26], nausea [22, 24, 26, 30, 32, 35], abnormal liver function [22, 24], headache and dizziness [26, 30], and the like. The results showed that experimental groups had no significant adverse events compared with the control groups.

### 3.10. Other Outcomes

Four trials [17, 22, 25, 32] reported heart rate of 335 patients. Tumor necrosis factor-*α* (TNF-*α*) and interleukin 6 (IL-6) were selected as outcome indexes in 2 studies [15, 30] with 228 patients. Only 1 trial [31] evaluated the indicators of troponin I (cTni) and hypersensitive C-reactive protein (hs-CRP), 1 trail [21] reported the levels of serum growth-promoting factor-1 (IGF-1) and troponin (cTnT), and 1 trail [27] evaluated the levels of angiotensin II (Ang II) and aldosterone (ALD) after treatment. One study reported the level of soluble growth stimulation expression gene 2 protein (sST2) [33]. The results showed that experimental groups significantly ameliorated the indicators compared with the control groups.

### 3.11. Publication Bias

**Figure 10:**
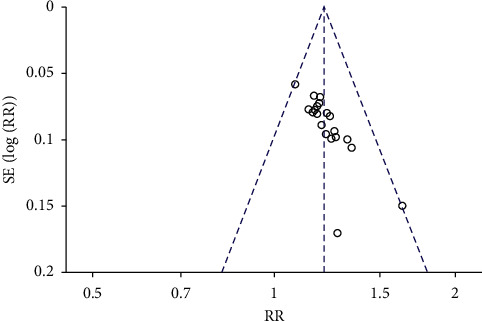
Funnel plot for the publication bias of the clinical efficacy.

Publication bias was expressed by the use of a funnel plot based on the data for clinical efficacy. Twenty studies [15–27, 29–35] were included in the funnel plot and are detailed in Figure 10. The analysis results showed that the bias funnel plot was asymmetrical, which indicated the possibility of publication bias.

## 4. Discussion

Chronic heart failure (CHF) is a major problem in modern medicine due to the incidence increases year by year, and the mortality rate increases sharply in recent years. In addition, CHF has a trend of being younger, and the 5-year survival rate is close to that of malignant tumors [36]. CHF is cardiac dysfunction caused by the failure of one or more cardiac chambers to maintain blood flow through the cardiac chambers, which has a significant negative impact on the quality of life and is a serious threat to patient health. The symptoms of CHF include chronic cough, rapid or irregular heartbeat, fatigue, fluid retention, and difficulty breathing [37]. The occurrence of CHF is related to age, with the prevalence rate of less than 2% in people under 60 years old, more than 14% in people aged 60–79 years old, and 28% in people over 80 years old affected by CHF [38]. At present, several therapeutic Western medicines are available to treat CHF that have improved survival, including diuretics, vasodilators, positive inotropic drugs, RAAS inhibitors, beta-blockers, and antiheart failure drugs. The long-term use of conventional Western medicines will have great side effects and the effect is not better, while traditional Chinese medicine in the treatment of chronic heart failure has the advantages of small side effects, multiple approaches, and multiple targets; so, it has been widely recognized by clinical medical workers.

Qishen Yiqi dropping pill mainly contains the ingredients of *Astragalus*, *Salvia miltiorrhiza*, *Panax notoginseng*, and *Dalbergia*. Modern pharmacology shows that *Astragalus* has the effects of reducing the cardiac load to dilate blood vessels, decreasing peripheral vascular resistance and inhibiting platelets, increasing the calcium inflow of cells, activating calmodulin, and reducing the breakdown of cyclic adenosine; thus, *Astragalus* reaches to improve the excitability of myocardium and produce a strong cardiac role [27]. In addition, the roles of *Astragalus* include antioxidant free radicals, increasing the antioxidant capacity of myocardium and LVEF, and inhibiting ventricular cell apoptosis [39]. Tanshinone is the main component of *Salvia miltiorrhiza*, which has the function of inhibiting platelet aggregation and anticoagulation besides increasing cardiac contractility and improving cardiac function indexes [40]. The main component of notoginseng is *Panax notoginseng* saponins, which plays the role of anticoagulation, dilating blood vessels, and improving coronary blood supply. *Dalbergia* can repair cell damage, promote angiogenesis, and reduce blood lipid and blood pressure [41].

The clinical efficacy is the most commonly used measure to evaluate the therapeutic efficacy in patients. In this study, the clinical effective rates of experimental groups were 92.3%, significantly higher than 76.3% of the control group. Both LVEDD and LVESD are the indexes of cardiac function, and LVEF can be stable and reliable in reflecting left ventricular function. The analysis results showed that Qishen Yiqi dropping pill combined with conventional Western medicine could increase the left ventricular ejection fraction and significantly improve the LVEDD, LVESD, and other indicators, suggesting that these effects of Qishen Yiqi dropping pill combined with conventional Western medicine may be the basis for the treatment of chronic heart failure. The 6 min walking distance objectively reflects the exercise tolerance of patients with CHF and then reflects the cardiac function. BNP is a quantitative marker of heart failure. The levels of BNP were high during left/right ventricular dysfunction, so they were reliable indicators to judge CHF [42]. The meta-analysis demonstrated that, compared with conventional Western medicine alone, Qishen Yiqi dropping pill combined with conventional Western medicine significantly increased the 6 min walking distance and decreased the levels of BNP of patients with CHF. In summary, Qishen Yiqi dropping pill combined with conventional Western medicine effectively improved the cardiac function of CHF and then proceeded to the next step to improve the quality of life of patients.

Although the clinical efficacy and safety of Qishen Yiqi dropping pill combined with conventional Western medicine in the treatment of CHF had undergone a large number of trials and rigorous methodological analysis, the existence of publication bias suggested that this study still had limitations. First, this study included 21 research literatures and finally, all of which were in Chinese, and most of the included literatures were small sample size studies with low quality. Second, the intervention measures and treatment course of each trial were not identical, which led to the great heterogeneity of each trial. With regard to methodological quality, it must be noted that both the blinding of participants and personnel and blinding of outcome assessment were not reported in any of the trials. In addition, none of the included literatures reported the specific grouping scheme. Finally, no serious adverse reactions occurred during the treatment, and whether there will be serious adverse reactions after long-term use still needs a lot of clinical research studies because the observation time was too short. Therefore, it is necessary to carry out a large sample clinical trial, which is randomly double-blind and scientifically designed to evaluate the long-term effect, so as to further verify the safety and reliability of Qishen Yiqi dropping pill combined with conventional Western medicine in the treatment of CHF in the future research.

## 5. Conclusions

In summary, this systematic review suggested that Qishen Yiqi dropping pill combined with conventional Western medicine provide an obvious clinical efficacy for the treatment of CHF, indicating that the therapy has some clinical potential. However, due to the small samples and generally lower quality studies included in this review, we expect more evidence from high-quality trials to confirm the advantages of the extensive clinical use of Qishen Yiqi dropping pill combined with conventional Western medicine for patients with CHF.

## Figures and Tables

**Table 1:** Characteristics of included studies.

The first author (years)	Total cases (E/C)	Sex, M/F	Age (*y*), range (mean)	Course of disease (*y*) range (mean)	NYHA	Duration	Intervention measures	Outcome indicators
Chen (2017)	152 (76/76)	C: 40/36	C: 44–81 (57.75 ± 7.52)	NR	C: II20, III31, IV25	4 W	C: CT 25 mg/time, 2 times/d	①②③⑧
		E: 42/34	E: 47–84 (56.64 ± 7.32)		E: II18, III30, IV28		E: C + 0.5 gQ/time, 3 times/d	⑨

Hu (2019)	82 (41/41)	C: 29/12	C: 42–70 (59.89 ± 6.02)	NR	NR	2 M	C: CT 7 mg/time, 2 times/d	①②③④
		E: 26/15	E: 47–84 (60.34 ± 7.21)				E: C + 0.5 gQ/time, 3 times/d	⑨

Jiang (2019)	84 (42/42)	C: 20/22	C: 50–75 (56.55 ± 5.95)	NR	C: I-II17, III20, IV5	3 M	C: CT 50 mg/time, 2 times/d	①④⑥⑧
		E: 25/17	E: 50–75 (57.43 ± 6.45)		E: I-II16, III18, IV8		E: C + 0.7 gQ/time, 3 times/d	—

Jin (2019)	85 (45/40)	C: 25/15	C: 44–75 (58.72 ± 5.22)	NR	III57, IV28	2 M	C: CT 20 mg/time, 3 times/d	①②③④
		E: 25/20	E: 45–72 (57.46 ± 5.15)				E: C + 0.5 gQ/time, 3 times/d	⑤⑦⑨

Li (2017)	94 (47/47)	C: 27/20	C: 59.14 ± 4.97	C: 5.53 ± 0.74	C: III32, IV15	8 W	C: CT 20 mg/time, 3 times/d	①②③④
		E: 29/18	E: 58.36 ± 5.39	E: 5.41 ± 0.83	E: III31, IV16		E: C + 0.5 gQ/time, 3 times/d	⑤⑨

Li (2018)	210 (105/105)	C: 57/48	C: 50–70 (62.35 ± 6.86)	C: 2–5 (2.31 ± 0.37)	C: II32, III47, IV26	3 M	C: CT 20 mg/time, 3 times/d	①②③④
		E: 63/42	E: 52–68 (62.44 ± 6.73)	E: 2–5 (2.29 ± 0.41)	E: II34, III46, IV25		E: C + 0.5 gQ/time, 3 times/d	⑤

Liu (2019)	178 (79/79)	98/80	55–78 (64.52 ± 6.67)	NR	NR	1 W	C: CT 12.5 mg/time, 3 times/d	①②③④
							E: C + 0.5 gQ/time, 3 times/d	⑥⑨

Wu (2016)	160 (80/80)	C: 38/42	C: 50–85 (63.4 ± 9.4)	C: 4–21 (10.6 ± 4.9)	C: III61, IV19	8 W	C: CT	①④⑤⑦
		E: 36/44	E: 52–86 (63.2 ± 13.6)	E: 3–22 (10.1 ± 5.1)	E: III64, IV16		E: C + 0.5 gQ/time, 3 times/d	⑧⑨

Xin (2015)	80 (40/40)	NR	NR	NR	NR	8 W	C: CT	①⑥⑦⑨
							E: C + 0.5 gQ/time, 3 times/d	—

Xu (2017)	146 (76/70)	C: 38/32	C: 52–84 (63.8 ± 12.2)	C: 3–18 (10.1 ± 5.2)	C: III37, IV33	8 W	C: CT	①④⑤⑦
		E: 42/34	E: 53–82 (62.4 ± 11.8)	E: 2–17 (9.5 ± 4.3)	E: III40, IV36		E: C + 0.5 gQ/time, 3 times/d	⑨

Xu (2019)	30 (15/15)	C: 9/6	C: 54–79 (64.27 ± 3.41)	C: 2–18 (10.36 ± 5.27)	C: III14, IV1	1 M	C: CT 20 mg/time, 1 time/d	①④⑤⑦
		E: 7/8	E: 53–78 (64.19 ± 3.46)	E: 2–17 (10.26 ± 5.37)	E: III13, IV2		E: C + 0.5 gQ/time, 2 times/d	⑧

Zeng (2019)	98 (49/49)	NR	C: 74.92 ± 11.08	NR	NR	3 M	C: CT 2.5–20 mg/time, 3 times/d	①②③④
			E: 72.47 ± 9.89				E: C + 0.5 gQ/time, 3 times/d	⑥⑨

Zhang (2017)	96 (48/48)	C: 29/19	C: 51–74 (61.34 ± 2.84)	C: 2–13 (7.18 ± 1.36)	C: III31, IV17	3 M	C: CT 20 mg/time, 3 times/d	①②③④
		E: 26/22	E: 50–73 (64.19 ± 3.46)	E: 2–12 (7.23 ± 1.32)	E: III29, IV19		E: C + 0.5 gQ/time, 3 times/d	⑤⑦⑧⑨

Zhang (2017)	86 (44/42)	C: 21/21	C: 62.1 ± 9.1	C: 3.7 ± 3.1	C: II23, III12, IV7	3 M	C: CT 20 mg/time, 3 times/d	④⑦
		E: 19/25	E: 57.8 ± 6.7	E: 4.6 ± 2.7	E: II19, III17, IV8		E: C + 0.5 gQ/time, 3 times/d	—

Zhang (2018)	80 (40/40)	C: 25/15	C: 47–75 (55.17 ± 5.62)	C: 1–8 (5.87 ± 0.76)	C: III26, IV14	8 W	C: CT 20 mg/time, 3 times/d	①②③④
		E: 23/17	E: 45–73 (54.32 ± 5.26)	E: 2–9 (5.61 ± 0.83)	E: III27, IV13		E: C + 0.5 gQ/time, 3 times/d	—

Zhao (2017)	90 (45/45)	C: 22/23	C: 65.23 ± 10.64	C: 6.57 ± 3.37	C: II16, III18, IV11	3 M	C: CT 20 mg/time, 3 times/d	①②③④
		E: 20/25	E: 64.07 ± 11.37	E: 7.05 ± 4.23	E: II13, III20, IV12		E: C + 0.5 gQ/time, 3 times/d	⑤⑧⑨

Zou (2019)	76 (38/38)	C: 20/18	C: 46–75 (61.52 ± 5.81)	C: 1–6 (2.99 ± 0.83)	C: I8, II8, III11, IV11	4 W	C: CT 20 mg/time, 1 time/d	①②③④
		E: 21/17	E: 46–75 (61.38 ± 5.71)	E: 1–6 (2.915 ± 0.76)	E: I9, II7, III9, IV13		E: C + 0.5 gQ/time, 3 times/d	⑤⑧⑨

Ye (2020)	64 (32/32)	C: 21/11	C: 58–76 (63.7 ± 14.1)	C: 3–8 (5.0 ± 2.1)	C: I-II20, III12	3 M	C: CT 50 mg/time, 2 times/d	①⑥⑧⑨
		E: 20/12	E: 51–78 (64.5 ± 15.3)	E: 3–8 (5.5 ± 1.8)	E: I-II22, III10		E: C + 0.52 gQ/time, 3 times/d	—

An (2020)	90 (45/45)	C: 29/16	C: 53–74 (57.85 ± 3.73)	NR	NR	8 W	C: CT 10 mg/time, 2 times/d	①④⑥⑧
		E: 33/12	E: 54–72 (58.12 ± 2.25)				E: C + 0.5 gQ/time, 3 times/d	—

Li (2020)	98 (49/49)	C: 28/21	C: 51–76 (63 ± 6)	NR	NR	12 W	C: CT 10 mg/time, 2 times/d	①⑥
		E: 26/23	E: 49–79 (63 ± 6)				E: C + 0.5 gQ/time, 3 times/d	—

Zhang (2020)	83 (42/41)	C: 24/17	C: 62.42 ± 8.60	NR	C: II23, III14, IV4	3 M	C: CT	①④⑤⑨
		E: 27/15	E: 65.07 ± 8.42		E: II20, III16, IV6		E: C + 0.5 gQ/time, 3 times/d	—

Notes: C, control group; E, experimental group; F, female; M, male; NR, not report; NYHA, New York Heart Association; CT, conventional treatment; Q, Qishen Yiqi dropping pill; W, weeks; M, months. Outcome indicators (① clinical efficacy rate; ②LVESD; ③LVEDD; ④LVEF; ⑤BNP; ⑥NT-proBNP; ⑦6-MWD; ⑧other indicators; ⑨adverse reactions).

**Table 2:** The side effects of included trails.

The first author (year)	Experimental group	Control group
Chen (2017)	0	0
Hu (2019)	0	0
Jiang (2019)	NR	NR
Jin (2019)	0	0
Li (2017)	0	0
Li (2018)	NR	NR
Liu (2019)	4/89 (4.49%)	3/89 (3.37%)
Wu (2016)	3/80 (3.75%)	3/80 (3.75%)
Xin (2015)	0	0
Xu (2017)	3/76 (3.95%)	4/70 (5.71%)
Xu (2019)	NR	NR
Zeng (2019)	7/49 (16.7%)	7/49 (16.7%)
Zhang (2017)	0	1/48 (2.08%)
Zhang (2017)	NR	NR
Zhang (2018)	0	0
Zhao (2017)	5/45 (11.1%)	3/45 (6.70%)
Zou (2019)	4/38 (10.53%)	3/38 (7.89%)
Zhang (2020)	6/42 (14.29%)	5/41 (12.20%)
An (2020)	0	1/32 (3.13%)
Ye (2020)	NR	NR
Li (2020)	NR	NR

Notes: NR, not report.

## Data Availability

The data used to support the findings of this study are available from the corresponding author upon request.
